# Influence of Fatigue on Tackling Ability in Rugby League Players: Role of Muscular Strength, Endurance, and Aerobic Qualities

**DOI:** 10.1371/journal.pone.0163161

**Published:** 2016-10-31

**Authors:** Tim J. Gabbett

**Affiliations:** Gabbett Performance Solutions, Brisbane, Australia; Universidade de Tras-os-Montes e Alto Douro, PORTUGAL

## Abstract

This study investigated the influence of repeated high-intensity effort exercise on tackling ability in rugby league players, and determined the relationship between physical qualities and tackling ability under fatigued conditions in these athletes. Eleven semi-professional rugby league players underwent measurements of speed (10 m and 40 m sprint), upper-body strength (4 repetition maximum [RM] bench press and weighted chin-up), upper-body muscular endurance (body mass maximum repetition chin-up, body mass maximum repetition dips), lower-body strength (4RM squat), and estimated maximal aerobic power (multi-stage fitness test). Tackling ability was assessed using a standardized one-on-one tackling test, before, during, and following four bouts of repeated high-intensity effort (RHIE) exercise. The relationship between physical qualities and fatigue-induced decrements in tackling ability were determined using Pearson product moment correlation coefficients. Each cycle of the RHIE protocol induced progressive reductions in tackling ability. A moderate reduction (Effect Size = ~-1.17 ± 0.60, -34.1 ± 24.3%) in tackling ability occurred after the fourth cycle of the RHIE protocol. Players with greater relative lower-body strength (i.e. 4RM squat/kg) had the best tackling ability under fatigued conditions (r = 0.72, p = 0.013). There were no significant relationships between tackling ability under fatigued conditions and any other physical quality. These findings suggest that lower-body strength protects against fatigue-induced decrements in tackling ability. The development of lower-body strength should be a priority to facilitate the development of robust tackling skills that are maintained under fatigue.

## Introduction

Success in collision sports (such as rugby union, rugby league, and American football) is dependent, at least in part, on tackling ability, the ability to tolerate physical collisions, and the ability to ‘win’ the tackle contest [1–4]. Due to the large number of injuries that occur during tackles and collisions [5–7], the tackle contest has received considerable attention in these sports. Although obvious similarities in the tackle contest exist between American football, rugby union, and rugby league, the majority of investigations on tackling ability in collision sports has been performed on rugby league players. Gabbett and Ryan [2] demonstrated that a standardized one-on-one tackle assessment could discriminate between professional and semi-professional rugby league players. Furthermore, higher-skilled tacklers made a greater proportion of dominant tackles and missed a smaller proportion of tackles than lesser-skilled tacklers [2]. These findings demonstrate the practical utility of a standardized tackle assessment to identify talent, while also providing information predictive of tackling performance in competition.

Several studies have investigated the physical qualities associated with tackling ability in junior and senior rugby league players competing at both an elite and sub-elite level [8–10]. Gabbett et al [9] reported that age (r = 0.41), playing experience (r = 0.70), skinfold thickness (r = -0.59), acceleration (r = 0.41) and lower-body muscular power (r = 0.38) were associated with tackling ability in professional rugby league players. Similar results were found in junior players; greater acceleration and lower-body muscular power qualities were associated with better tackling ability [10]. A recent study of semi-professional rugby league players also demonstrated that better upper- and lower-body strength and upper-body power were associated (r = 0.70–0.96) with superior tackling ability [11]. Moreover, training-induced improvements in lower-body strength were also associated with improvements in tackling ability (r = 0.60) [12]. Collectively these findings demonstrate the importance of well-developed physical qualities to tackling ability in rugby league players.

Fatigue, induced by repeated high-intensity exercise (RHIE) has been shown to reduce tackling ability in rugby league players [13]. Interestingly, players with the best tackling ability in a non-fatigued state demonstrated the greatest decrement in tackling ability under fatigued conditions. In addition, a significant association was observed between aerobic power (r = -0.62) and change of direction speed (r = 0.68) and fatigue-induced decrements in tackling ability, suggesting that strength and conditioning programs designed to develop endurance, and change of direction speed may reduce fatigue-induced decrements in tackling ability. While this study provided important information about the effect of fatigue on tackling ability, and the physical qualities that are important to maintaining tackling ability under fatigued conditions, some limitations warrant discussion. Firstly, as the study was performed on amateur rugby league players, the degree to which the findings relate to players of greater ability is unclear. Secondly, only a limited number of physical qualities were measured; no assessment of upper- or lower-body strength was performed.

It is possible that stronger players use a lower proportion of their maximum strength to execute tackles than relatively weaker players [14]. Consequently, well-developed upper- and lower-body strength could protect against fatigue-induced decrements in tackling ability. Gabbett and Wheeler [15] recently developed a game-specific RHIE test for rugby league players. Players with well-developed acceleration and upper-body muscular endurance qualities were better able to maintain physical performance over the duration of the RHIE test [15]. While this study provided important information on the correlates of RHIE ability in rugby league players, such a test also provides an ideal platform to investigate the influence of fatigue, induced by RHIE activity, on technical skill performance. With this in mind, the purpose of this study was two-fold: (1) to investigate the influence of game-specific, repeated high-intensity effort exercise on tackling ability in rugby league players, and (2) to determine the relationship between selected physical qualities (i.e. speed, upper- and lower-body strength, upper-body muscular endurance, and aerobic power) and tackling ability under fatigued conditions in these athletes.

## Materials and Methods

### Subjects

Eleven rugby league players (mean ± SD age, 24 ± 3 yr) participated in this study. Subjects were hit-up forwards from the same semi-professional rugby league team. Hit-up forwards were selected due to the high contact and repeated high-intensity effort (RHIE) demands of this position [16]. At the time of the study, players had completed 8 weeks of pre-season training, were in good physical condition, and were free from injury. All participants received a clear explanation of the study, including the risks and benefits, and written consent was obtained. All experimental procedures were approved by the institutional review board for human investigation at the Australian Institute of Sport (#20070614).

### Fitness Testing Battery

The tests described in this study are similar to those performed in previous investigations [15]. Although data were collected on players of similar quality to those described previously [15], the emphasis of this study was to (i) determine the influence of fatigue, elicited through game-specific RHIE activity on tackling skill, and (ii) determine which, if any physical qualities, protected against fatigue-induced reductions in tackling ability.

Players underwent fitness testing over a two-week period in February as part of their pre-season training program for the forthcoming playing season. Testing was conducted two weeks before the first competition match. The physical tests performed were (i) 10 m and 40 m sprint, (ii) 4 repetition maximum (RM) bench press, (iii) 4RM squat, (iv) 4RM weighted chin-up, (v) 4RM dips, (vi) body mass maximum repetition chin-up, (vii) body mass maximum repetition dips, and (viii) multi-stage fitness test. All testing was conducted at the same time of day (~ 6.00 pm). Participants were requested to abstain from strenuous physical exercise for 72 hours before testing, with only light skills, and minimal strength and conditioning performed in this period. Players performed no physical activity in the 24 hours prior to testing. Players were also instructed to consume their normal pre-training diet and to ensure adequate hydration at the time of testing.

#### Speed

The running speed of players was evaluated with a 10 m and 4 0m sprint effort using dual beam electronic timing gates (Swift Performance Equipment, New South Wales, Australia). The timing gates were positioned 10 m and 40 m cross wind from a pre-determined starting point. Players were instructed to run as quickly as possible along the 40 m distance from a standing start. Speed was measured to the nearest 0.01 s with the fastest value obtained from three trials used as the speed score. The intraclass correlation coefficient for test-retest reliability and typical error of measurement for the 10 m and 40 m sprint tests were 0.95 and 0.97, and 1.8% and 1.2%, respectively.

#### Upper-body muscular strength

Maximum upper-body strength was assessed using a 4RM bench press exercise and weighted chin-up. For the bench press, athletes used a free-weight Olympic-style barbell. Players lowered the bar on to the chest, and were required to fully extend their arms for the lift to be counted as a valid trial. Players were not permitted to bounce the bar off their chest and the feet were required to remain in contact with the ground, and buttocks in contact with the bench for the trial to be considered valid.

The 4RM weighted chin-up was added to the testing battery to assess the strength of the shoulder extensors. The 4RM weighted chin-up was determined by adding the athlete’s body mass to an additional mass which was attached to the athlete. The chin-up test was performed with a supinated grip and begun with an eccentric phase. The trial was considered successful if the athlete fully extended their arms in the eccentric phase and was able to pull their body upwards to return to the starting position.

#### Upper-body muscular endurance

Players’ strength-endurance was assessed using maximum repetition chin-up and dip tests. Using their body mass as resistance, athletes were encouraged to perform as many repetitions as possible until fatigue.

#### Lower-body muscular strength

Maximum lower-body strength was assessed using a 4RM full-squat exercise performed using a free-weight Olympic-style barbell. After warming up with progressively heavier loads, the athlete attempted their 4RM. Players were required to lower their body so that their thighs were past parallel with the floor, and fully extend the hip and knee joints for the trial to be considered valid.

#### Maximal aerobic power

Maximal aerobic power was estimated using the multi-stage fitness test [17]. Players were required to run back and forth (i.e. shuttle run) along a 20m track, keeping in time with a series of signals on a compact disk. The frequency of the audible signals (and hence, running speed) was progressively increased, until volitional exhaustion was reached. Maximal aerobic power ($\dot{\text{V}}{\text{O}}_{2}$ max) was estimated using regression equations described by Ramsbottom et al [17].

### Repeated High-Intensity Effort Protocol

The RHIE protocol, which has been described in detail previously [15] was developed based on the most extreme physical demands of rugby league match-play [16, 18]. The RHIE protocol consisted of (i) 1 x 10 m sprint (performed on a 6 s cycle), (ii) 3 x full contact 1-on-1 tackling efforts on a similar sized partner (the ball-carrier wore a chest shield, with each tackle lasting 4 s, and performed on a 6 s cycle), and (iii) a 30 m active recovery (jog ~13 s) back to the starting position. Players were provided a 3 s count-down before performing the next repetition. Players were required to perform 4 repetitions of the protocol, with each repetition completed in 40 s. For each contact effort, the tackler was required to move a distance of no further than 3 m (approximate distance travelled when making a tackle from marker), and the ball-carrier was instructed to run aggressively, and at speed towards the tackler [15].

Video footage was taken from the rear, side, and front of the defending player during the RHIE protocol. Tackling technique was objectively assessed by a sport scientist using standardized technical criteria. The technical criteria were developed by two expert coaches who also used these criteria as cues when coaching tackling technique in rugby league players.

The technical criteria included:

1. Contacting the target in the centre of gravity
2. Initial contact made with the shoulder
3. Body position square and aligned
4. Leg drive upon contact
5. Watching the target onto the shoulder
6. Centre of gravity forward of base of support

Players were awarded one point for each occasion they achieved the relevant criteria and a score of zero if they failed to achieve the criteria. A total score (out of 6, and reported in arbitrary units) was awarded for each tackle, with this score also reported as a percentage.

### Statistical Analysis

Differences in tackling ability across the repeated-effort protocol, and ratings of perceived exertion after each cycle of the protocol were compared using Cohen’s effect size (ES) statistic [19]. Effect sizes of <0.2, 0.2–0.6, 0.61–1.2, 1.21–2.0, and >2.0 were considered trivial, small, moderate, large, and very large, respectively [20]. Magnitudes of differences among the four cycles were classified as substantially greater or lesser difference when there was a ≥75% likelihood of the effect being equal to or greater than the smallest worthwhile change estimated as 0.2 x between-subject standard deviation (small ES). Effects with less certainty were classified as trivial and where ±90% CI of the ES crossed the boundaries of ES -0.2 and 0.2, the effect was reported as unclear [20]. The relationship between selected physical qualities (i.e. speed, upper and lower body strength, upper-body muscular endurance, and aerobic power) and fatigue-induced decrements in tackling ability were determined using Pearson product moment correlation coefficients ±95% CI. The level of significance was set at p<0.05 and all data are reported as mean ± SD.

## Results

### Tackling Ability under Fatigue

The physical qualities of the players are presented in Table 1.

**Table 1:** Physical qualities of the semi-professional rugby league players.

Age (yr)	24.6 ± 2.7
Body Mass (kg)	100.0 ± 11.4
*Speed*	
10 m Sprint (s)	1.83 ± 0.04
40 m Sprint (s)	5.49 ± 0.18
*Absolute Strength*	
4RM Bench Press (kg)	126.7 ± 15.3
4RM Chin-Up (kg)	27.1 ± 9.9
4RM Squat (kg)	171.3 ± 16.4
*Relative Strength*	
4RM Bench Press (kg.^-1^.kg^-1^)	1.21 ± 0.12
4RM Chin-Up (kg.^-1^.kg^-1^)	0.30 ± 0.09
4RM Squat (kg.^-1^.kg^-1^)	1.68 ± 0.15
*Upper-Body Muscular Endurance*	
Maximum Repetition Chin-Up (no.)	14.0 ± 5.9
Maximum Repetition Dips (no.)	22.3 ± 6.5
*Maximal Aerobic Power*	
Estimated $\dot{\text{V}}{\text{O}}_{2}$ max (ml.kg.^-1^.min^-1^)	52.1 ± 3.0

Data are mean ± SD. $\dot{\text{V}}{\text{O}}_{2}$ max = maximal aerobic power. RM = repetition maximum.

**Fig 1:**
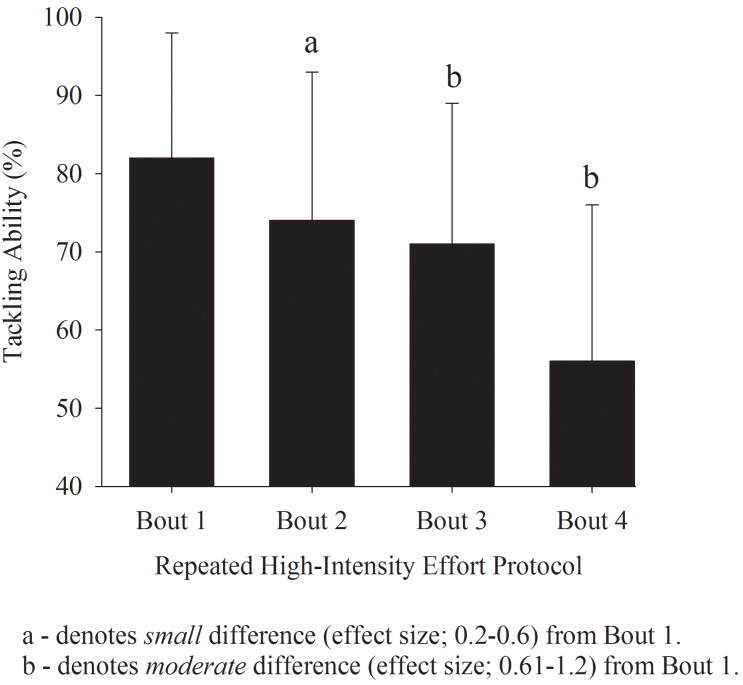
Influence of repeated high-intensity exercise on tackling ability in rugby league players. Data are expressed as percentage changes ± 90% confidence intervals from the original score.

Each cycle of the RHIE protocol induced progressive reductions in tackling ability. Small reductions in tackling ability occurred after the first (ES = ~-0.44 ± 0.74, -10.4 ± 19.4%, 81%, likely) and second (ES = ~0.60 ± 0.72, -14.5 ± 21.3%, 90%, likely) RHIE bout. A moderate reduction (ES = ~-1.17 ± 0.60, -34.1 ± 24.3%, 100%, likely) in tackling ability occurred after the fourth cycle of the RHIE protocol (Fig 1).

### Correlates of Tackling Ability under Fatigue

Players with the best tackling ability under pre-fatigue conditions had the best tackling ability under fatigued conditions (r = 0.84, p = 0.001) (Table 2). Players with greater relative lower-body strength (i.e. 4RM squat/kg) had the best tackling ability under fatigued conditions (r = 0.72, p = 0.013). There were no significant relationships between tackling ability under fatigued conditions and any other physical quality (Table 3).

**Table 2:** Relationship among *absolute* physical qualities and tackling ability under fatigued conditions in semi-professional rugby league players.

	TackA^Rest^	TackA^Fat^	Bench^ABS^	Chins^ABS^	Squat^ABS^	Chins^Max^	Dips^Max^	Sprint^10^	Sprint^40^
TackA^Rest^	1.00								
	-								
TackA^Fat^	0.84^†^	1.00							
	0.48–0.96	-							
Bench^ABS^	0.36	0.11	1.00						
	-0.31–0.79	-0.52–0.67	-						
Chins^ABS^	0.24	0.09	-0.08	1.00					
	-0.42–0.73	-0.54–0.66	-0.65–0.55	-					
Squat^ABS^	0.46	0.52	0.34	0.01	1.00				
	-0.19–0.83	-0.12–0.85	-0.33–0.78	-0.59–0.61	-				
Chins^Max^	0.29	0.23	0.06	0.86^†^	-0.10	1.00			
	-0.38–0.76	-0.54–0.66	-0.56–0.64	0.54–0.96	-0.66–0.53	-			
Dips^Max^	0.34	0.32	0.46	0.20	-0.26	0.59	1.00		
	-0.33–0.78	-0.76–0.36	-0.19–0.83	-0.45–0.71	-0.74–0.40	-0.02–0.88	-		
Sprint^10^	-0.46	-0.18	-0.90^†^	-0.13	-0.38	-0.24	-0.46	1.00	
	-0.83–0.19	-0.7–0.5	-0.65–-0.97	-0.68–0.51	-0.78–0.28	-0.73–0.42	-0.83–0.19	-	
Sprint^40^	-0.42	-0.38	-0.70*	-0.05	-0.68*	-0.20	-0.30	0.82^†^	1.00
	-0.81–0.24	-0.80–0.28	-0.17–-0.92	-0.63–0.57	-0.14–-0.91	-0.71–0.45	-0.76–0.37	0.43–0.95	-

Data are reported as Pearson product moment correlation coefficients, r ±95% CI.

* Denotes significance at P<0.05.

^†^ Denotes significance at P<0.01.

TackA^Rest^ = tackling ability under non-fatigued conditions;

TackA^Fat^ = tackling ability under fatigued conditions;

Bench^ABS^ = 4RM bench press;

Chins^ABS^ = 4RM chin-up;

Squat^ABS^ = 4RM squat;

Chins^Max^ = maximum repetition chin-ups with body mass as resistance;

Dips^Max^ = maximum repetition dips with body mass as resistance;

Sprint^10^ = 10 m sprint;

Sprint^40^ = 40 m sprint.

**Table 3:** Relationship among *relative* physical qualities (expressed relative to body mass) and tackling ability under fatigued conditions in semi-professional rugby league players.

	TackA^Rest^	TackA^Fat^	Bench^REL^	Chins^REL^	Squat^REL^	$\dot{\text{V}}{\text{O}}_{2}$ max
TackA^Rest^	1.00					
	-					
TackA^Fat^	0.84^†^	1.00				
	0.48–0.96	-				
Bench^REL^	0.47	0.32	1.00			
	-0.18–0.83	-0.35–0.77	-			
Chins^REL^	-0.01	0.02	0.31	1.00		
	-0.61–0.59	-0.59–0.61	-0.36–0.77	-		
Squat^REL^	0.68*	0.72*	0.26	0.14	1.00	
	0.13–0.91	0.19–0.92	-0.74–0.40	-0.50–0.68	-	
$\dot{\text{V}}{\text{O}}_{2}$ max	0.24	0.16	0.41	0.35	0.63*	1.00
	-0.42–0.73	-0.48–0.69	-0.25–0.81	-0.32–0.78	0.05–0.89	-

Data are reported as Pearson product moment correlation coefficients, r ±95% CI.

* Denotes significance at P<0.05.

^†^ Denotes significance at P<0.01.

TackA^Rest^ = tackling ability under non-fatigued conditions;

TackA^Fat^ = tackling ability under fatigued conditions;

Bench^REL^ = 4RM bench press expressed relative to body mass;

Chins^REL^ = 4RM chin-up expressed relative to body mass;

Squat^REL^ = 4RM squat expressed relative to body mass;

$\dot{\text{V}}{\text{O}}_{2}$ max = maximal aerobic power.

## Discussion

This is the first study to investigate tackling ability of rugby league players prior to, and under fatigued conditions, and determine the relationship between tackling ability and selected physical qualities such as speed, upper- and lower-body strength, upper-body muscular endurance, and aerobic power. The results of this study demonstrate small to moderate reductions in tackling ability under fatigued conditions. Players with greater relative squat capabilities demonstrated better tackling ability under fatigued conditions. These findings suggest that lower-body strength protects against fatigue-induced decrements in tackling ability, and that the development of lower-body strength should be a priority to facilitate the development of robust tackling skills that are maintained under fatigue.

The reductions in tackling ability with progressively increasing fatigue in the semi-professional rugby league players of this study, is consistent with the findings from amateur players [13]. The reductions in tackling ability occurred as a result of failing to maintain a square and aligned body position, watch the target onto the shoulder, and make contact with the centre of gravity forward of the base of support. Failing to maintain leg drive upon contact was the criteria most influenced by fatigue. Driving the legs after contact is an important technical requirement for tackling success. In rugby union, the chances of a ball-carrier breaking the tackle decreased by 80% when the tackler performed a leg drive [4]. These findings suggest that reductions in tackling ability under fatigued conditions occur due to limitations in technical, physical and perceptual qualities. While making contact with the centre of gravity and leg drive upon contact can be readily improved through technical instruction and appropriate strength training, it is likely that some improvements can also be made through perceptual training. Indeed, simply training the player to watch the target onto the shoulder is likely to improve the tackling player’s ability to meet more of the technical criteria associated with a quality tackle.

We found a significant association between relative lower-body strength and tackling ability under fatigued conditions; players with better squat per kilogram of body mass also had better tackling performances under fatigue. It is likely that stronger players use a lower proportion of their maximum strength to execute tackles than relatively weaker players [14, 21]. Furthermore, the strong association between lower-body strength and tackling ability under fatigue most likely reflects the similar movement patterns between activities; forceful hip and knee extension is common for both the squat and the leg drive action in tackling. Conversely, we failed to find an association between upper-body endurance and tackling ability under fatigued conditions. These findings have important implications for strength and conditioning programs that underpin the ability of players to perform technical skills. Firstly, our findings demonstrate that lower-body strength protects against fatigue-induced decrements in tackling ability. Secondly, upper-body muscular endurance and aerobic fitness appear to have no effect on the ability of semi-professional rugby league players to maintain tackling ability under fatigued conditions.

The importance of lower-body strength to tackling ability under fatigued conditions is consistent with previous studies that have shown that well-developed lower-body strength contributes to team selection [22], greater RHIE ability during matches [22], and enhanced recovery following matches [23]. Greater maximal squatting ability has also been shown to be positively associated with tackling ability and the proportion of dominant tackles performed during match-play [24]. These findings suggest that lower-body strength underpins effective tackling performance under game-specific fatigue, and as such, strength programs should emphasize the development of this physical quality in rugby league players. However, it should be noted that a significant correlation does not imply cause and effect; further studies are required to determine if improvements in lower-body strength attenuate the decrements in tackling ability in response to game-specific fatigue. Equally, any statistical association is best suited to the population from which it is derived. It is therefore possible that physical qualities in addition to, or other than lower-body strength may share a greater association with tackling ability under fatigued conditions in rugby league players of different (higher- or lower) skill levels. Finally, while we found no associations between upper-body strength and tackling ability, it is possible that different tackling strategies (e.g. ‘over-the-ball’ contact and ‘catching’ the ball-carrier) may rely more on upper-body strength and therefore, cannot be dismissed as a potentially important physical quality for different variations of tackling skill.

### Practical Applications

There are several findings from this study that have relevance to the applied sport scientist and strength and conditioning coach. Firstly, we provide the expected decrement in tackling ability (~-34%) in response to game-specific repeated-effort exercise in semi-professional rugby league players. Secondly, and importantly, we identify key physical qualities that protect against these fatigue-induced reductions in skill. Relative lower-body strength is an important physical quality that underpins effective tackling ability under physiological fatigue. Practicing tackling skills during repeated-effort exercise may allow for better transfer of skills under game-specific fatigue. Equally importantly, the development of lower-body strength, rather than aerobic fitness or muscular endurance, may offer a more effective approach of preventing reductions in tackling ability under fatigued conditions. Speculatively, integrating post-activation potentiation approaches (e.g. combining heavy squat training with specific tackling drills) may offer a novel method of transferring lower-body strength developed in the gymnasium to ‘real-life’ tackling skills.

In conclusion, this study investigated the tackling ability of rugby league players prior to, and under fatigued conditions, and determined the relationship between tackling ability and speed, upper- and lower-body strength, upper-body muscular endurance, and aerobic power. The results of this study demonstrate that game-specific repeated–effort exercise elicits reductions in tackling ability in rugby league players. Players with greater relative lower-body strength demonstrated better tackling ability under fatigued conditions. These findings suggest that lower-body strength protects against fatigue-induced decrements in tackling ability and that the development of lower-body strength should be a priority to facilitate the development of robust tackling skills that are maintained under fatigue.

## Supporting Information

S1 File (PDF)

S2 File (PDF)
