# Supplementary material for: Influence of Fatigue on Tackling Ability in Rugby League Players: Role of Muscular Strength, Endurance, and Aerobic Qualities
Source: PLoS One. 2016 Oct 31;11(10):e0163161. doi: 10.1371/journal.pone.0163161 (PMC5087954; doi:10.1371/journal.pone.0163161)
Supplement: S1 File — (PDF) [file pone.0163161.s001.pdf]

Subject 1	Cycle 1	Cycle 2	Cycle 3	Cycle 4	Total
Contact in COG	1	1	1	1	4
Contact with Shoulder	1	1	0	0	2
Body position Square/Aligned	1	1	0	0	2
Leg Drive Upon Contact	0	0	1	0	1
Watch Target onto Shoulder	1	1	0	0	2
COG forward of base	1	1	1	1	4
**Total**	**5**	**5**	**3**	**2**	15
**Total (%)**	**83**	**83**	**50**	**33**	63

Subject 2	Cycle 1	Cycle 2	Cycle 3	Cycle 4	Total
Contact in COG	0	0	0	0	0
Contact with Shoulder	1	0	1	1	3
Body position Square/Aligned	1	1	1	1	4
Leg Drive Upon Contact	0	1	1	1	3
Watch Target onto Shoulder	1	0	1	0	2
COG forward of base	1	1	1	1	4
**Total**	**4**	**3**	**5**	**4**	16
**Total (%)**	**67**	**50**	**83**	**67**	67

Subject 3	Cycle 1	Cycle 2	Cycle 3	Cycle 4	Total
Contact in COG	1	1	1	0	3
Contact with Shoulder	1	0	1	1	3
Body position Square/Aligned	0	0	0	0	0
Leg Drive Upon Contact	1	1	1	0	3
Watch Target onto Shoulder	1	0	1	1	3
COG forward of base	1	1	1	0	3
**Total**	**5**	**3**	**5**	**2**	15
**Total (%)**	**83**	**50**	**83**	**33**	63

Subject 4	Cycle 1	Cycle 2	Cycle 3	Cycle 4	Total
Contact in COG	1	1	1	1	4
Contact with Shoulder	1	1	1	1	4
Body position Square/Aligned	1	1	1	1	4
Leg Drive Upon Contact	1	1	1	1	4
Watch Target onto Shoulder	1	1	1	1	4
COG forward of base	1	1	1	1	4
**Total**	**6**	**6**	**6**	**6**	24
**Total (%)**	**100**	**100**	**100**	**100**	100

Subject 5	Cycle 1	Cycle 2	Cycle 3	Cycle 4	Total
Contact in COG	0	1	0	0	1
Contact with Shoulder	1	1	1	1	4
Body position Square/Aligned	1	1	1	0	3
Leg Drive Upon Contact	1	0	0	1	2
Watch Target onto Shoulder	1	1	1	0	3
COG forward of base	1	1	1	0	3
**Total**	**5**	**5**	**4**	**2**	16
**Total (%)**	**83**	**83**	**67**	**33**	67

Subject 6	Cycle 1	Cycle 2	Cycle 3	Cycle 4	Total
Contact in COG	1	1	1	1	4
Contact with Shoulder	1	1	1	1	4
Body position Square/Aligned	0	1	0	0	1
Leg Drive Upon Contact	1	0	1	0	2
Watch Target onto Shoulder	0	0	0	0	0
COG forward of base	1	1	1	1	4
**Total**	**4**	**4**	**4**	**3**	15
**Total (%)**	**67**	**67**	**67**	**50**	63

Subject 7	Cycle 1	Cycle 2	Cycle 3	Cycle 4	Total
Contact in COG	1	1	1	1	4
Contact with Shoulder	1	1	1	1	4
Body position Square/Aligned	1	1	1	0	3
Leg Drive Upon Contact	0	0	0	0	0
Watch Target onto Shoulder	1	1	1	1	4
COG forward of base	1	0	0	1	2
**Total**	**5**	**4**	**4**	**4**	17
**Total (%)**	**83**	**67**	**67**	**67**	71

Subject 8	Cycle 1	Cycle 2	Cycle 3	Cycle 4	Total
Contact in COG	0	0	0	0	0
Contact with Shoulder	1	1	1	1	4
Body position Square/Aligned	0	0	0	0	0
Leg Drive Upon Contact	1	0	0	0	1
Watch Target onto Shoulder	0	1	1	1	3
COG forward of base	1	1	0	1	3
**Total**	**3**	**3**	**2**	**3**	11
**Total (%)**	**50**	**50**	**33**	**50**	46

Subject 9	Cycle 1	Cycle 2	Cycle 3	Cycle 4	Total
Contact in COG	1	1	0	1	3
Contact with Shoulder	1	1	1	1	4
Body position Square/Aligned	1	1	0	1	3
Leg Drive Upon Contact	1	1	1	0	3
Watch Target onto Shoulder	1	1	1	0	3
COG forward of base	1	1	1	1	4
**Total**	**6**	**6**	**4**	**4**	20
**Total (%)**	**100**	**100**	**67**	**67**	83

Subject 10	Cycle 1	Cycle 2	Cycle 3	Cycle 4	Total
Contact in COG	1	1	1	1	4
Contact with Shoulder	1	1	1	1	4
Body position Square/Aligned	1	1	1	1	4
Leg Drive Upon Contact	1	0	0	0	1
Watch Target onto Shoulder	1	1	1	0	3
COG forward of base	1	1	1	0	3
**Total**	**6**	**5**	**5**	**3**	19
**Total (%)**	**100**	**83**	**83**	**50**	79

Subject 11	Cycle 1	Cycle 2	Cycle 3	Cycle 4	Total
Contact in COG	1	1	1	1	4
Contact with Shoulder	1	1	1	1	4
Body position Square/Aligned	1	1	1	0	3
Leg Drive Upon Contact	0	0	0	0	0
Watch Target onto Shoulder	1	1	1	1	4
COG forward of base	1	1	1	1	4
**Total**	**5**	**5**	**5**	**4**	19
**Total (%)**	**83**	**83**	**83**	**67**	79

Subject 1	Cycle 1	Cycle 2	Cycle 3	Cycle 4	Total
Contact in COG	100%	100%	100%	100%	4
Contact with Shoulder	100%	100%	0%	0%	2
Body position Square/Aligned	100%	100%	0%	0%	2
Leg Drive Upon Contact	0%	0%	100%	0%	1
Watch Target onto Shoulder	100%	100%	0%	0%	2
COG forward of base	100%	100%	100%	100%	4
**Total**	**5**	**5**	**3**	**2**	15
**Total (%)**	**83**	**83**	**50**	**33**	63

Subject 2	Cycle 1	Cycle 2	Cycle 3	Cycle 4	Total
Contact in COG	0.0%	0.0%	0.0%	0.0%	0
Contact with Shoulder	100.0%	0.0%	100.0%	100.0%	3
Body position Square/Aligned	100.0%	100.0%	100.0%	100.0%	4
Leg Drive Upon Contact	0.0%	100.0%	100.0%	100.0%	3
Watch Target onto Shoulder	100.0%	0.0%	100.0%	0.0%	2
COG forward of base	100.0%	100.0%	100.0%	100.0%	4
**Total**	**4**	**3**	**5**	**4**	16
**Total (%)**	**67**	**50**	**83**	**67**	67

Subject 3	Cycle 1	Cycle 2	Cycle 3	Cycle 4	Total
Contact in COG	100.0%	100.0%	100.0%	0.0%	3
Contact with Shoulder	100.0%	0.0%	100.0%	100.0%	3
Body position Square/Aligned	0.0%	0.0%	0.0%	0.0%	0
Leg Drive Upon Contact	100.0%	100.0%	100.0%	0.0%	3
Watch Target onto Shoulder	100.0%	0.0%	100.0%	100.0%	3
COG forward of base	100.0%	100.0%	100.0%	0.0%	3
**Total**	**5**	**3**	**5**	**2**	15
**Total (%)**	**83**	**50**	**83**	**33**	63

Subject 4	Cycle 1	Cycle 2	Cycle 3	Cycle 4	Total
Contact in COG	100.0%	100.0%	100.0%	100.0%	4
Contact with Shoulder	100.0%	100.0%	100.0%	100.0%	4
Body position Square/Aligned	100.0%	100.0%	100.0%	100.0%	4
Leg Drive Upon Contact	100.0%	100.0%	100.0%	100.0%	4
Watch Target onto Shoulder	100.0%	100.0%	100.0%	100.0%	4
COG forward of base	100.0%	100.0%	100.0%	100.0%	4
**Total**	**6**	**6**	**6**	**6**	24
**Total (%)**	**100**	**100**	**100**	**100**	100

Subject 5	Cycle 1	Cycle 2	Cycle 3	Cycle 4	Total
Contact in COG	0.0%	100.0%	0.0%	0.0%	1
Contact with Shoulder	100.0%	100.0%	100.0%	100.0%	4
Body position Square/Aligned	100.0%	100.0%	100.0%	0.0%	3
Leg Drive Upon Contact	100.0%	0.0%	0.0%	100.0%	2
Watch Target onto Shoulder	100.0%	100.0%	100.0%	0.0%	3
COG forward of base	100.0%	100.0%	100.0%	0.0%	3
**Total**	**5**	**5**	**4**	**2**	16
**Total (%)**	**83**	**83**	**67**	**33**	67

Subject 6	Cycle 1	Cycle 2	Cycle 3	Cycle 4	Total
Contact in COG	100.0%	100.0%	100.0%	100.0%	4
Contact with Shoulder	100.0%	100.0%	100.0%	100.0%	4
Body position Square/Aligned	0.0%	100.0%	0.0%	0.0%	1
Leg Drive Upon Contact	100.0%	0.0%	100.0%	0.0%	2
Watch Target onto Shoulder	0.0%	0.0%	0.0%	0.0%	0
COG forward of base	100.0%	100.0%	100.0%	100.0%	4
**Total**	**4**	**4**	**4**	**3**	15
**Total (%)**	**67**	**67**	**67**	**50**	63

Subject 7	Cycle 1	Cycle 2	Cycle 3	Cycle 4	Total
Contact in COG	100.0%	100.0%	100.0%	100.0%	4
Contact with Shoulder	100.0%	100.0%	100.0%	100.0%	4
Body position Square/Aligned	100.0%	100.0%	100.0%	0.0%	3
Leg Drive Upon Contact	0.0%	0.0%	0.0%	0.0%	0
Watch Target onto Shoulder	100.0%	100.0%	100.0%	100.0%	4
COG forward of base	100.0%	0.0%	0.0%	100.0%	2
**Total**	**5**	**4**	**4**	**4**	17
**Total (%)**	**83**	**67**	**67**	**67**	71

Subject 8	Cycle 1	Cycle 2	Cycle 3	Cycle 4	Total
Contact in COG	0.0%	0.0%	0.0%	0.0%	0
Contact with Shoulder	100.0%	100.0%	100.0%	100.0%	4
Body position Square/Aligned	0.0%	0.0%	0.0%	0.0%	0
Leg Drive Upon Contact	100.0%	0.0%	0.0%	0.0%	1
Watch Target onto Shoulder	0.0%	100.0%	100.0%	100.0%	3
COG forward of base	100.0%	100.0%	0.0%	100.0%	3
**Total**	**3**	**3**	**2**	**3**	11
**Total (%)**	**50**	**50**	**33**	**50**	46

Subject 9	Cycle 1	Cycle 2	Cycle 3	Cycle 4	Total
Contact in COG	100.0%	100.0%	0.0%	100.0%	3
Contact with Shoulder	100.0%	100.0%	100.0%	100.0%	4
Body position Square/Aligned	100.0%	100.0%	0.0%	100.0%	3
Leg Drive Upon Contact	100.0%	100.0%	100.0%	0.0%	3
Watch Target onto Shoulder	100.0%	100.0%	100.0%	0.0%	3
COG forward of base	100.0%	100.0%	100.0%	100.0%	4
**Total**	**6**	**6**	**4**	**4**	20
**Total (%)**	**100**	**100**	**67**	**67**	83

Subject 10	Cycle 1	Cycle 2	Cycle 3	Cycle 4	Total
Contact in COG	100.0%	100.0%	100.0%	100.0%	4
Contact with Shoulder	100.0%	100.0%	100.0%	100.0%	4
Body position Square/Aligned	100.0%	100.0%	100.0%	100.0%	4
Leg Drive Upon Contact	100.0%	0.0%	0.0%	0.0%	1
Watch Target onto Shoulder	100.0%	100.0%	100.0%	0.0%	3
COG forward of base	100.0%	100.0%	100.0%	0.0%	3
**Total**	**6**	**5**	**5**	**3**	19
**Total (%)**	**100**	**83**	**83**	**50**	79

Subject 11	Cycle 1	Cycle 2	Cycle 3	Cycle 4	Total
Contact in COG	100.0%	100.0%	100.0%	100.0%	4
Contact with Shoulder	100.0%	100.0%	100.0%	100.0%	4
Body position Square/Aligned	100.0%	100.0%	100.0%	0.0%	3
Leg Drive Upon Contact	0.0%	0.0%	0.0%	0.0%	0
Watch Target onto Shoulder	100.0%	100.0%	100.0%	100.0%	4
COG forward of base	100.0%	100.0%	100.0%	100.0%	4
**Total**	**5**	**5**	**5**	**4**	19
**Total (%)**	**83**	**83**	**83**	**67**	79
