# Supplementary material for: Influence of Fatigue on Tackling Ability in Rugby League Players: Role of Muscular Strength, Endurance, and Aerobic Qualities
Source: PLoS One. 2016 Oct 31;11(10):e0163161. doi: 10.1371/journal.pone.0163161 (PMC5087954; doi:10.1371/journal.pone.0163161)
Supplement: S2 File — (PDF) [file pone.0163161.s002.pdf]

Subject	Cycle 1	Cycle 2	Cycle 3	Cycle 4	Total Score	Decrement (%)	Age	Body Mass	Bench Press (10RM)	Bench Press (4RM)
1	83	83	50	33	63	-25.0	27.6	89.0	110.6	126.7
2	67	50	83	67	67	0.0	24.6	100.0	105	120
3	83	50	83	33	63	-25.0	25.2	103.0	100	120
4	100	100	100	100	100	0.0	24.6	93.0	90	105
5	83	83	67	33	67	-20.0	24.6	130.0	140	150
6	67	67	67	50	63	-6.3	23.9	98.0	100	110
7	83	67	67	67	71	-15.0	20.0	98.0	120	140
8	50	50	33	50	46	-8.3	20.7	103.0	95	110
9	100	100	67	67	83	-16.7	25.4	100.0	125	145
10	100	83	83	50	79	-20.8	29.7	86.0	110.6	126.7
11	83	83	83	67	79	-5.0	24.3	100.0	120	140
							**24.60**	**100.00**	**110.56**	**126.67**
							**2.70**	**11.37**	**14.74**	**15.33**
**Correlations (with first score)**							0.565	-0.233	0.296	0.369
**Correlations (with total score)**							0.322	-0.271	0.060	0.115
**95% CI (LOWER)**							-0.35	-0.75	-0.56	-0.52
**95% CI (UPPER)**							0.77	0.39	0.64	0.67

Squats (10RM)	Squats (4RM)	Chins (4RM)	Chins (Max)	Dips (4RM)	Dips (Max)	Hang Clean (1RM)	MSFT	Yo-Yo	10m Sprint	40m Sprint
143.9	171.3	27.1	14	41.7	22.3	96.1	51.4	13.51	1.82	5.45
160	180	12.5	8	40	15	110	51.4	14.5	1.86	5.46
140	180	40	14	40	10	90	54	14.3	1.84	5.59
140	180	27.1	14	25	22.3	90	51.9	14.3	1.89	5.65
180	171.3	8	2	50	22.3	110	52.1	14.3	1.8	5.5
120	150	27.1	14	40	22.3	75	47.7	14.3	1.85	5.55
140	180	40	22	45	28	100	59	15.54	1.8	5.39
115	140	27.1	14	40	22.3	90	49.6	13.52	1.88	5.85
140	160	35	24	35	36	100	49.3	14.3	1.78	5.35
143.9	171.3	27.1	14	41.7	22.3	96.1	52.1	14.3	1.8	5.5
160	200	27.1	14	60	22.3	100	54.3	14.3	1.77	5.13
**143.89**	**171.26**	**27.10**	**14.00**	**41.67**	**22.28**	**96.11**	**52.07**	**14.29**	**1.83**	**5.49**
**18.14**	**16.39**	**9.87**	**5.87**	**8.66**	**6.52**	**9.94**	**3.01**	**0.53**	**0.04**	**0.18**
0.338	0.469	0.242	0.289	-0.203	0.341	0.183	0.249	0.270	-0.456	-0.418
0.275	0.517	0.094	0.240	-0.305	0.329	0.118	0.163	0.336	-0.182	-0.384
-0.39	-0.12	-0.54	-0.42	-0.76	-0.34	-0.52	-0.48	-0.33	-0.7	-0.8
0.75	0.85	0.66	0.73	0.36	0.78	0.67	0.69	0.78	0.47	0.28

Bench Press/BW	Squats/BW	Chins/BW	Dips/BW	Hang Clean/BW	Ideal	Actual
1.2	1.7	0.3	0.4	0.9	83	63
1.2	1.7	0.3	0.4	0.9	67	67
1.2	1.7	0.4	0.4	0.9	83	63
1.1	1.9	0.3	0.3	1.0	100	100
1.2	1.7	0.1	0.4	0.8	83	67
1.1	1.5	0.3	0.4	0.8	67	63
1.4	1.8	0.4	0.5	1.0	83	71
1.1	1.4	0.3	0.4	0.9	50	46
1.5	1.6	0.3	0.4	1.0	100	83
1.2	1.7	0.3	0.4	0.9	100	79
1.2	1.7	0.3	0.4	0.9	83	79
**1.21**	**1.68**	**0.30**	**0.39**	**0.90**	**81.82**	**70.83**
**0.12**	**0.15**	**0.09**	**0.05**	**0.07**	**15.73**	**14.19**
0.467	0.680	-0.005	-0.409	0.531		
0.321	0.707	0.021	-0.616	0.536		
-0.35	0.19	-0.59	-0.89	-0.09		
0.77	0.92	0.61	-0.02	0.86		
